# Comparing ischemic cardiovascular effectiveness and safety between individual SGLT-2 inhibitors and DPP-4 inhibitors in patients with type 2 diabetes: a nationwide population-based cohort study

**DOI:** 10.3389/fphar.2024.1443175

**Published:** 2024-10-30

**Authors:** Hayeon Kim, Jun-Ho Seo, Jin Hyun Nam, Yejee Lim, Kyung Hee Choi, Kyungim Kim

**Affiliations:** ^1^ College of Pharmacy, Korea University, Sejong, Republic of Korea; ^2^ Department of Big Data Science, Korea University, Sejong, Republic of Korea; ^3^ Division of Big Data Science, Korea University, Sejong, Republic of Korea; ^4^ Department of Internal Medicine, Seoul National University Bundang Hospital, Seoul National University College of Medicine, Seongnam, Republic of Korea; ^5^ College of Pharmacy, Gachon University, Incheon, Republic of Korea; ^6^ Institute of Pharmaceutical Science, Korea University, Sejong, Republic of Korea

**Keywords:** sodium-glucose transporter 2 inhibitors, empagliflozin, dapagliflozin, dipeptidyl-peptidase IV inhibitors, cardiovascular events, retrospective study

## Abstract

**Objectives:**

This study compared the ischemic cardiovascular events (iCVEs) effectiveness and safety of initiating empagliflozin or dapagliflozin with those of dipeptidyl peptidase-4 inhibitors (DPP-4is), as well as the comparative effects between empagliflozin and dapagliflozin.

**Methods:**

Using data from the National Health Insurance Service in Korea, patients with type 2 diabetes mellitus (T2DM) who were newly prescribed empagliflozin, dapagliflozin, or DPP-4is from 2016 to 2019 and who did not have a recent CVE history were included. A Cox proportional hazards regression model was used to estimate the adjusted hazard ratio (aHR) with 95% confidence intervals (CIs) for iCVEs and safety events.

**Results:**

Empagliflozin and dapagliflozin significantly reduced the risks of ischemic stroke (aHR 0.568, 95% CI 0.408–0.791; aHR 0.612, 95% CI 0.476–0.786, respectively) and all-cause mortality (aHR 0.590, 95% CI 0.442–0.788; aHR 0.730, 95% CI 0.603–0.884, respectively) compared with DPP-4is. Initiating dapagliflozin or empagliflozin was associated with significantly lower incidence of severe hypoglycemia, bone fracture, urinary tract infection, and acute kidney injury than that of DPP-4is. No significant differences were observed between empagliflozin and dapagliflozin in iCVEs and most safety outcomes.

**Conclusion:**

Empagliflozin and dapagliflozin showed significant preventive effects on ischemic stroke and all-cause mortality compared with DPP-4is in patients with T2DM, and their protective effects were similar. Both empagliflozin and dapagliflozin were not related to the harmful effects on most safety events. These results suggest that it may be beneficial to initiate empagliflozin or dapagliflozin for ischemic stroke prevention in patients with T2DM. However, further validation studies, such as randomized controlled trials, are needed to generalize these results.

## 1 Introduction

Diabetes, one of the leading causes of death and disability worldwide, is a serious and chronic disease that affects more than 10% of the world’s adult population (International Diabetes Federation, 2021). Patients with diabetes are susceptible to several macrovascular/microvascular complications, such as cardiovascular disease, nephropathy, neuropathy, retinopathy, and peripheral vascular disease (International Diabetes Federation, 2021). Among these, cardiovascular disease is the principal cause of death in patients with diabetes, especially type 2 diabetes mellitus (T2DM) (Morrish et al., 2001; Einarson et al., 2018). These characteristics of diabetes place a great economic and psychological burden not only on the individual with diabetes but also on their family, healthcare systems, and society. Therefore, it has become critical to prevent cardiovascular disease in patients with T2DM, beyond simply controlling the blood glucose levels. Sodium–glucose cotransporter-2 inhibitors (SGLT-2is) have been shown to have favorable effects in reducing the risk of a major adverse cardiac event (MACE), which is the composite outcome including cardiovascular death, nonfatal myocardial infarction (MI), or nonfatal stroke, through large randomized controlled trials (RCTs) and real-world studies (Zinman et al., 2015; Kosiborod et al., 2017; Neal et al., 2017; Kosiborod et al., 2018; Wiviott et al., 2019). SGLT-2is are currently recommended for patients who have established atherosclerotic cardiovascular disease (ASCVD) or are at high risk of ASCVD to reduce MACE (ElSayed et al., 2023a).

However, there are still some research gaps that warrant further investigation regarding the effects of SGLT-2is on cardiovascular events (CVEs). Several early representative RCTs demonstrating the salutary cardioprotective effects of SGLT-2is, including EMPA-REG (Zinman et al., 2015), CANVAS (Neal et al., 2017), and DECLARE–TIMI 58 (Wiviott et al., 2019) compared SGLT-2is with the placebo group. Although recent studies comparing SGLT-2is with active controls using real-world data have consistently reported a reduced risk of hospitalization for heart failure in SGLT-2is, results on ischemic cardiovascular events (iCVEs), such as unstable angina and MI, are insufficient (Kosiborod et al., 2017; Kim et al., 2018; Kosiborod et al., 2018; Persson et al., 2018; Dawwas et al., 2019; Han et al., 2021; Patorno et al., 2022). And real-world studies on the effects of SGLT-2is on stroke have produced inconsistent results (Kosiborod et al., 2018; Persson et al., 2018; Dawwas et al., 2019; Han et al., 2021; Kashiwagi et al., 2022; Patorno et al., 2022). Therefore, despite the cumulative evidence on SGLT-2is, the effect of SGLT-2is on iCVEs and stroke in patients with T2DM compared with active controls remains controversial.

In addition, previous studies have limitations in that they examined the class effects of SGLT-2is on iCVEs rather than individual drugs (Kosiborod et al., 2017; Kim et al., 2018; Kosiborod et al., 2018; Dawwas et al., 2019; Han et al., 2021; Kashiwagi et al., 2022). However, each drug of the SGLT-2i class may have different effects on the human body because its structure and properties, including the selectivity to SGLT-2/SGLT-1 receptors, are slightly different (Isaji, 2011; Pahud de Mortanges et al., 2021). This was also observed in previous comparative studies reporting glycosylated hemoglobin (HbA1c) and fasting blood glucose between canagliflozin, empagliflozin, and dapagliflozin (Ku et al., 2019; Blonde et al., 2021; Ku et al., 2021).

Therefore, the primary objective of this study was to evaluate iCVEs and safety events associated with initiating empagliflozin or dapagliflozin, compared with dipeptidyl peptidase-4 inhibitors (DPP-4is) in patients with T2DM without a recent CVE history. The secondary objective was to compare the effects of empagliflozin and dapagliflozin on iCVEs and safety events.

## 2 Materials and methods

### 2.1 Data source

This is a population-based retrospective cohort study using data from the National Health Insurance Service (NHIS) database in South Korea. The NHIS is a universal mandatory single-payer healthcare coverage system that covers 97% of the Korean population. It provides de-identified longitudinal health-related records of individuals, such as sociodemographic data, diagnoses (International Classification of Disease, 10th Revision [ICD-10], code), therapeutic procedures, drug prescriptions (prescription date, days of supply, and dose and route of administration), and type of medical use (outpatient, inpatient, or emergency department). The structures of the NHIS dataset have been described in another publication (Cheol Seong et al., 2017). The NHIS has an annual or biennial health screening dataset comprising lifestyle questionnaire surveys, physical examination assessment, and laboratory test results. The current study used the claims and national health screening dataset provided by the NHIS. This study was approved by the Institutional Review Board of Korea University (KUIRB-2021-0036-01) and the Korea NHIS National Health Information Data Request Review Committee. All procedures were performed in accordance with relevant regulations and guidelines. The need for informed consent was waived.

### 2.2 Study population

**FIGURE 1:**
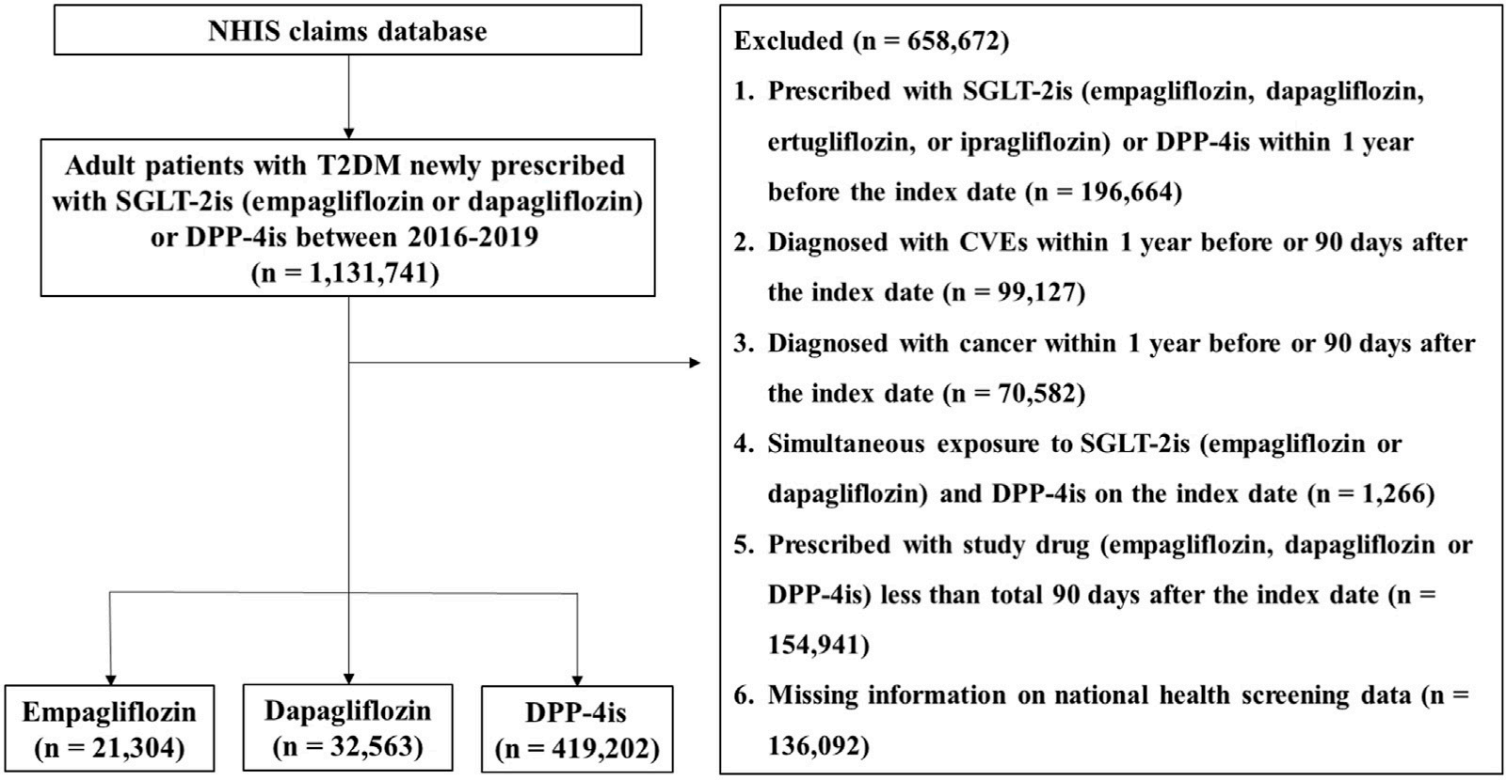
Flow diagram of the study population Abbreviations: NHIS, National Health Insurance Service; T2DM, type 2 diabetes mellitus; SGLT-2is, sodium–glucose cotransporter-2 inhibitors; DPP-4is, dipeptidyl peptidase-4 inhibitors; CVEs, cardiovascular events.

From the NHIS database, adult patients with T2DM aged 20–100 years who were newly prescribed empagliflozin, dapagliflozin, or DPP-4is (alogliptin, anagliptin, evogliptin, teneligliptin, linagliptin, saxagliptin, sitagliptin, vildagliptin, or gemigliptin) from 2016 to 2019 were selected. T2DM was defined if an individual had a T2DM diagnostic code (ICD-10 code, E11) as primary diagnosis or sub-diagnosis and at least one antidiabetic drug prescription history. DPP-4is were chosen as an active comparator rather than other antidiabetics for two reasons. First, DPP-4is show a similar ability to SGLT-2is in reducing HbA1c, with a difference of only approximately 0.09%–0.1% between the two classes (Scheen, 2020; Shao et al., 2020; ElSayed et al., 2023b). DPP-4is have no effect on CVEs such as MACE, MI, and stroke (ElSayed et al., 2023a), allowing us to evaluate the intrinsic benefits of SGLT-2is on iCVEs rather than the pathway of iCVEs reduction via glycemic control. Second, DPP-4is and SGLT-2is are widely used as second-line oral antidiabetic agents, making them relevant and meaningful “real-world” comparators. Patients who received a prescription for empagliflozin, dapagliflozin, or any DPP-4is as an initial or add-on medication were considered new users. The initiation date of the study drug was defined as the index date. To sort new users, patients who had been prescribed any SGLT-2is (empagliflozin, dapagliflozin, ertugliflozin, or ipragliflozin) or DPP-4is within 1 year before the index date were excluded. To minimize the impact of a previous CVE history on study outcomes, we excluded patients diagnosed with cardiovascular diseases such as MI, unstable angina, heart failure, or ischemic stroke or received coronary revascularization (percutaneous coronary intervention or coronary artery bypass graft) within 1 year before the index date or within 90 days after the index date. This is based on studies reporting that most recurrent CVEs occur within 1 year after the first event in patients with diabetes (van der Heijden et al., 2013; Berghout et al., 2023). Additionally, patients were excluded from this study if they had any of the following: a history of cancer within 1 year before the index date or within 90 days after the index date, simultaneous exposure to SGLT-2is (empagliflozin or dapagliflozin) and DPP-4is on the index date, a total duration of study drug prescription of <90 days after the index date, or unavailable health screening data. The detailed selection process of the study population is presented in Figure 1.

### 2.3 Study outcomes and follow-up period

The iCVE effectiveness outcome was composite iCVEs of MI (I21–I23), unstable angina (I20), or any procedure regarding coronary revascularization (percutaneous coronary intervention or coronary artery bypass graft). Each component of composite iCVEs, ischemic stroke (I63), and all-cause mortality were also evaluated. For safety outcomes, bone fracture, genital infection, severe hypoglycemia, urinary tract infection (UTI), diabetic ketoacidosis (DKA), acute kidney injury (AKI), and hypotension were assessed. The detailed corresponding codes for safety outcomes are presented elsewhere (Supplementary Table S1). Patients were followed up from the index date to the occurrence of outcome of interest, discontinuation of the initial study drugs (add-on or switch to other study drug or not refilling a prescription of study drug for ≥90 consecutive days), death, or the end of the study (31 December 2019), whichever came first.

### 2.4 Covariates

The covariates used in this study were age, sex, calendar index year, household income, region of residence, Charlson comorbidity index (CCI) score, comorbidities, co-medications, body mass index (BMI), smoking status, and adjusted Diabetes Complications Severity Index (aDCSI) (Table 1). The age groups were classified as <65, ≥65–<75, and ≥75. The household income groups, which were initially divided into 20 classes (class 0, lowest income; class 20, highest income) in the NHIS database, were recategorized into 4 classes (low, classes 0–5; medium-low, classes 6–10; medium-high, classes 11–15; and high, classes 16–20). The regions of residence were grouped as urban (Seoul, Gyeonggi, Busan, Daegu, Incheon, Gwangju, Daejeon, and Ulsan) and rural (Gangwon, Chungbuk, Chungnam, Jeonbuk, Jeonnam, Gyeongbuk, Gyeongnam, and Jeju) areas. The patients’ CCI scores were estimated from their disease records using previously validated algorithms (Quan et al., 2005). Comorbidity was defined as at least one hospitalization or outpatient for more than twice with the corresponding diagnostic code within 1 year before the index date. The data of co-medications that have been associated with cardiovascular disease were collected when they were prescribed for ≥60 days within 1 year before the index date; antihypertensive agents, antihyperlipidemic agents, antiplatelet agents, anticoagulant agents, and antidiabetic agents. For concomitant antidiabetic agents, drug classes such as insulin, glucagon-like peptide-1 receptor agonists, alpha-glucosidase inhibitors, sulfonylureas, thiazolidinediones, metformin, and meglitinides were included. Details of comorbidities and co-medications are documented in Supplementary Tables S1, S2. BMI was categorized into three groups (18.5–24.9, 25.0–29.9, and ≥30 kg/m^2^). Smoking status was categorized as never smoker, former smoker, and current smoker. The Diabetes Complications Severity Index (DCSI) is an indicator that classifies complications of diabetes into seven categories (55 subcategories) and scores them according to severity (0, normal; 1, abnormal; and 2, severe abnormal) (Young et al., 2008). In this study, aDCSI, a validated adapted version of the DCSI that excludes laboratory data, was used as a covariate to adjust for diabetes severity of the patient (Chang et al., 2012). When scoring aDCSI, cerebrovascular and cardiovascular complications were not considered because they overlapped with the current study outcomes; thus, the total modified score ranged from 0 to 9 (Supplementary Table S3).

**TABLE 1:** Baseline characteristics of the study population.

Variables	Empagliflozin (n = 21,304)	Dapagliflozin (n = 32,563)	DPP-4is^a^ (n = 419,202)	SD
Sex				−0.05
Male	12,566 (58.98)	19,380 (59.52)	256,990 (61.30)	
Female	8,738 (41.02)	13,183 (40.48)	162,212 (38.70)	
Age (years)				0.29
<65	18,608 (87.35)	29,169 (89.58)	319,577 (76.23)	
65–74	2,212 (10.38)	2,838 (8.72)	71,599 (17.08)	
≥75	484 (2.27)	556 (1.71)	28,026 (6.69)	
Calendar index year				0.51
2016	1,887 (8.86)	6,488 (19.92)	110,079 (26.26)	
2017	5,573 (26.16)	8,341 (25.61)	115,243 (27.49)	
2018	6,694 (31.42)	9,186 (28.21)	111,160 (26.52)	
2019	7,150 (33.56)	8,548 (26.25)	82,720 (19.73)	
Household income^b^ ^,^ ^c^				0.03
1	4,431 (20.80)	6,662 (20.46)	91,309 (21.78)	
2	4,255 (19.97)	6,454 (19.82)	82,107 (19.59)	
3	5,546 (26.03)	8,541 (26.23)	108,506 (25.88)	
4	6,838 (32.10)	10,504 (32.26)	131,884 (31.46)	
Region of residence^c^ ^,^ ^d^				0.01
Urban	14,507 (68.10)	22,333 (68.58)	283,085 (67.53)	
Rural	6,792 (31.88)	10,220 (31.39)	135,987 (32.44)	
Comorbidity				
Hypertension	9,784 (45.93)	14,027 (43.08)	170,717 (40.72)	0.11
Dyslipidemia	11,372 (53.38)	16,255 (49.92)	186,705 (44.54)	0.18
Atrial fibrillation	180 (0.84)	213 (0.65)	2,654 (0.63)	0.02
Chronic kidney disease	96 (0.45)	90 (0.28)	2,021 (0.48)	0.03
Diabetic retinopathy	375 (1.76)	481 (1.48)	6,526 (1.56)	0.02
Diabetic neuropathy	694 (3.26)	822 (2.52)	12,032 (2.87)	0.04
Diabetic nephropathy	403 (1.89)	596 (1.83)	5,658 (1.35)	0.04
Rheumatoid arthritis	259 (1.22)	367 (1.13)	6,068 (1.45)	−0.02
Charlson comorbidity index				0.10
0	2,646 (12.42)	4,573 (14.04)	61,874 (14.76)	
1	5,844 (27.43)	9,016 (27.69)	116,517 (27.79)	
2	6,396 (30.02)	9,747 (29.93)	120,628 (28.78)	
≥3	6,418 (30.13)	9,227 (28.34)	120,183 (28.67)	
Co-medications				
Antihypertensive agents	9,768 (45.85)	13,939 (42.81)	174,488 (41.62)	0.09
Antihyperlipidemic agents	9,576 (44.95)	13,516 (41.51)	155,245 (37.03)	0.16
Antiplatelet agents	2,626 (12.33)	3,424 (10.52)	46,728 (11.15)	0.06
Anticoagulant agents	127 (0.60)	132 (0.41)	1,690 (0.40)	0.03
Antidiabetic agents^e^	7,872 (36.95)	10,789 (33.13)	133,776 (31.91)	0.11
Body mass index (kg/m^2^)				0.41
18.5–24.9	5,046 (23.69)	7,311 (22.45)	167,190 (39.88)	
25–29.9	10,402 (48.83)	15,872 (48.74)	193,094 (46.06)	
≥30	5,856 (27.49)	9,380 (28.81)	58,918 (14.05)	
Smoking status				0.08
Never smoker	10,987 (51.57)	16,721 (51.35)	214,414 (51.15)	
Former smoker	5,995 (28.14)	8,670 (26.63)	108,195 (25.81)	
Current smoker	4,322 (20.29)	7,172 (22.02)	96,593 (23.04)	
aDCSI score^f^				0.08
0	12,440 (58.39)	19,061 (58.54)	240,149 (57.29)	
1	6,240 (29.29)	9,626 (29.56)	122,885 (29.31)	
2	2,093 (9.82)	3,139 (9.64)	43,836 (10.46)	
3	455 (2.14)	628 (1.93)	10,297 (2.46)	
4	67 (0.31)	98 (0.30)	1,787 (0.43)	
5	9 (0.04)	11 (0.03)	229 (0.05)	
6	—	—	19 (0.00)	
aDCSI with complication categories				
Retinopathy	3,277 (15.38)	4,774 (14.66)	64,142 (15.30)	0.02
Nephropathy	2,278 (10.69)	3,927 (12.06)	44,856 (10.70)	−0.04
Neuropathy	3,585 (16.83)	5,126 (15.74)	75,444 (18.00)	0.03
Peripheral vascular disease	2,228 (10.46)	3,227 (9.91)	48,674 (11.61)	−0.04
Metabolic disease	68 (0.32)	105 (0.32)	1,617 (0.39)	−0.01
eGFR (mL/min/1.73 m^2^)^c,g^				0.14
<60	745 (3.50)	929 (2.85)	21,309 (5.08)	
60–89.9	9,252 (43.43)	13,869 (42.59)	191,723 (45.74)	
≥90	11,193 (52.54)	17,605 (54.06)	202,247 (48.25)	
LDL-C (mg/dL)^c^ ^,^ ^g^				0.22
<70	2,373 (11.14)	3,485 (10.70)	47,545 (11.34)	
70–99.9	3,363 (15.79)	5,389 (16.55)	74,172 (17.69)	
100–129.9	3,386 (15.89)	5,570 (17.11)	79,573 (18.98)	
130–159.9	2,322 (10.90)	4,054 (12.45)	56,772 (13.54)	
≥160	1,561 (7.33)	2,654 (8.15)	37,020 (8.83)	

Data are presented as numbers (%) or means ± standard deviations.

Abbreviations: DPP-4is, dipeptidyl peptidase-4 inhibitors; SD, standardized difference; aDCSI, adjusted Diabetes Complications Severity Index; eGFR, estimated glomerular filtration rate; LDL-C, low-density lipoprotein cholesterol.

^a^ The DPP-4is group included patients who were newly prescribed alogliptin, anagliptin, evogliptin, teneligliptin, linagliptin, saxagliptin, sitagliptin, vildagliptin, or gemigliptin as an initial or add-on medication.

^b^ The household income levels were divided into 20 classes (from the lowest income class 1 to the highest income class 20) and categorized into 4 groups (low, classes 0–5; medium-low, classes 6–10; medium-high, classes 11–15; and high, classes 16–20).

^c^ The percentages within each column do not add up to 100% because some data are unavailable.

^d^ The regions of residence were grouped into the urban (Seoul, Gyeonggi, Busan, Daegu, Incheon, Gwangju, Daejeon, and Ulsan) and rural (Gangwon, Chungcheongbuk, Chungcheongnam, Jeollabuk, Jeollanam, Gyeongsangbuk, Gyeongsangnam, and Jeju) areas.

^e^ Glucose-lowering drugs other than the current study drugs (empagliflozin, dapagliflozin, and DPP-4is) were considered as concomitant antidiabetic agents. The drug classes, such as insulin, glucagon-like peptide-1 receptor agonists, alpha-glucosidase inhibitors, sulfonylureas, thiazolidinediones, metformin, and meglitinides, were included as antidiabetic agents.

^f^ The aDCSI is an indicator that classifies complications of diabetes into seven categories (55 subcategories) and scores them according to severity (0, normal; 1, abnormal; and 2, severe abnormal). This study modified the aDCSI, by excluding the categories of cerebrovascular and cardiovascular complications, which overlap with the outcomes of interest in this study.

^g^ The eGFR and LDL-C were used as variables in the subgroup analysis, not covariates for adjusting the hazard ratio.

### 2.5 Statistical analysis

The baseline characteristics of new users of empagliflozin, dapagliflozin, and DPP-4is are presented as frequencies and percentages. The differences between the groups were estimated using standardized differences. The incidence rate of outcomes was calculated by 100 person-years, and the cumulative incidence of CVEs was estimated using the Kaplan–Meier method. The Cox proportional hazards model was used to estimate the hazard ratio (HR) and 95% confidence intervals (CIs) for iCVEs in the empagliflozin or dapagliflozin groups compared with the DPP-4i group. To address the imbalance between study groups, the adjusted HR (aHR) was calculated after adjusting for all variables listed in Table 1, except for estimated glomerular filtration rate (eGFR) and low-density lipoprotein cholesterol (LDL-C). They were not considered covariates because the corresponding covariates chronic kidney disease and dyslipidemia were included as comorbidities. All analyses were conducted using SAS (version 9.4; SAS Institute, Cary, NC, United States).

Subgroup analysis was performed for composite iCVEs by stratifying patients according to the age group, sex, BMI, eGFR, LDL-C, aDCSI score, use of antiplatelet agents and insulin, and proportion of days covered (PDC) (≥80%). The PDC was calculated as the total number of non-overlapping prescription days divided by the total follow-up period. Sensitivity analyses were conducted to ensure the robustness of the study results. First, subdistribution hazard model was adopted to address the possible competing risk of mortality. Second, only iCVE outcomes that occurred ≥1 year after the index date were counted. Additionally, to examine the impact of unmeasured confounders, an E-value was calculated. This indicates the minimum strength of association that an unmeasured confounder needs to have with both drug choice (exposure) and outcomes to fully explain the observed association (VanderWeele and Ding, 2017).

## 3 Results

### 3.1 Study population and characteristics

A total of 473,069 patients were newly prescribed SGLT-2is or DPP-4is from 2016 to 2019, of whom 21,304, 32,563, and 419,202 received empagliflozin, dapagliflozin, and DPP-4is, respectively (Figure 1). The baseline characteristics of the study population of each group are summarized in Table 1. The mean age of the study population was 55.9 years and older in the order of the DPP-4is, empagliflozin, and dapagliflozin groups. The mean follow-up periods were 513.84 ± 408.02 days (empagliflozin, 397.56 ± 317.96; dapagliflozin, 440.61 ± 365.59; and DPP-4is, 525.44 ± 413.66).

### 3.2 Preventive effects on iCVEs

**FIGURE 2:**
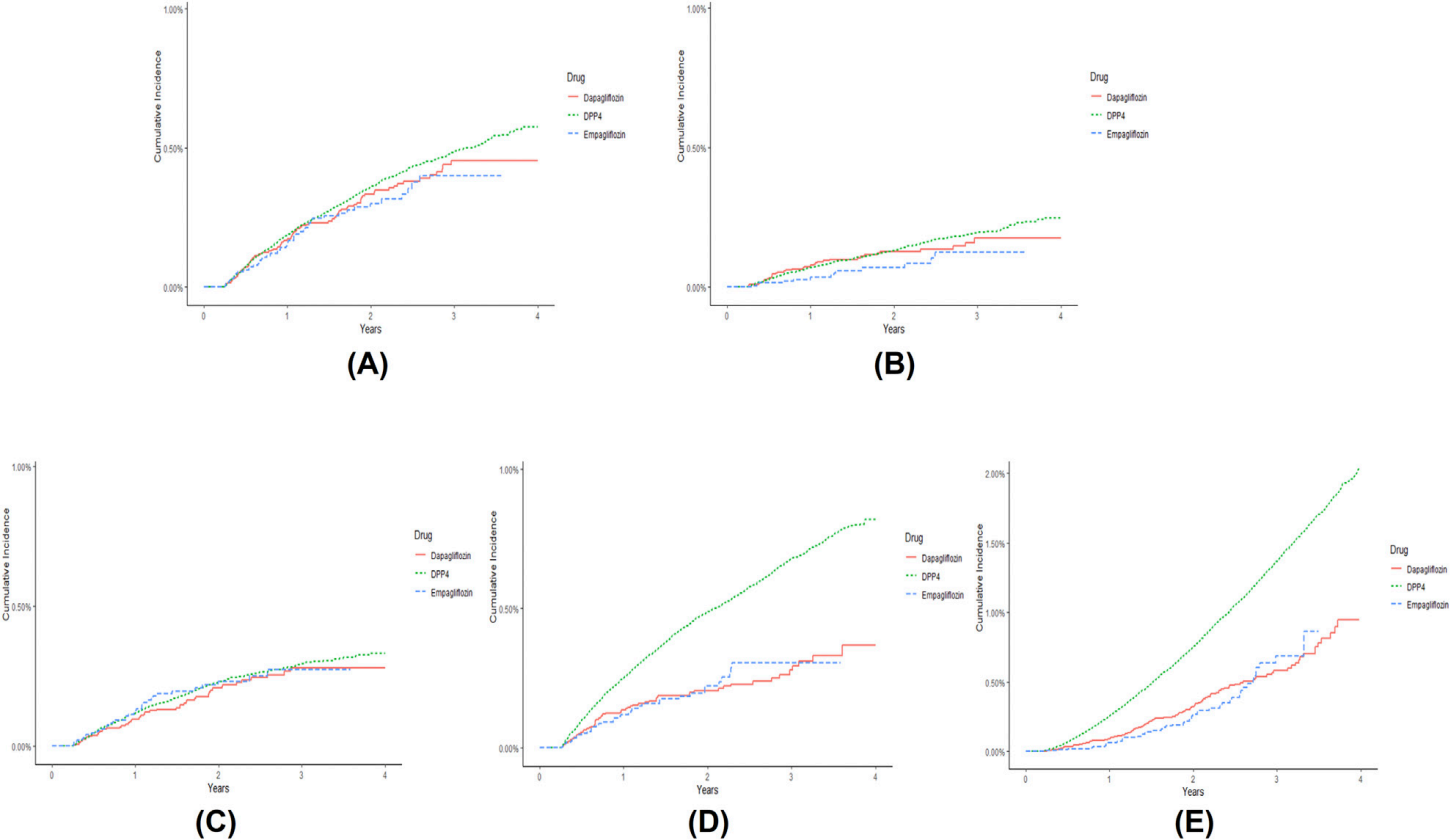
Cumulative incidence of **(A)** composite ischemic cardiovascular events (iCVEs), **(B)** myocardial infarction, **(C)** unstable angina, **(D)** ischemic stroke, and **(E)** all-cause mortality in patients newly prescribed empagliflozin (dashed line), dapagliflozin (solid line), and dipeptidyl peptidase-4 inhibitors (DPP-4is) (compact dashed line) over time. Composite iCVEs include myocardial infarction, unstable angina, or coronary revascularization. The cumulative incidence graph of coronary revascularization was omitted because few outcomes were identified.

The incidence rates of composite iCVEs were 0.14, 0.15, and 0.17 per 100 person-years in the empagliflozin, dapagliflozin, and DPP-4i groups, respectively. The aHR for composite iCVEs of the empagliflozin and dapagliflozin groups were not significantly different when compared with that of the DPP-4i group (aHR 0.925, 95% CI 0.694–1.233; aHR 1.019, 95% CI 0.823–1.262, respectively) (Table 2). No significant reduction in the risk of MI or unstable angina in both SGLT-2i initiating groups versus DPP-4is initiating group was observed. In contrast, both the new users of empagliflozin and dapagliflozin had a significantly lower risk of ischemic stroke than those of DPP-4is (aHR 0.568, 95% CI 0.408–0.791; aHR 0.612, 95% CI 0.476–0.786, respectively). The E-values for the point estimate and upper confidence limit were 2.918 and 1.842 for empagliflozin and 2.652 and 1.861 for dapagliflozin, respectively. Moreover, all-cause mortality was significantly lower in the empagliflozin and dapagliflozin new user groups than in the DPP-4i group (aHR 0.590, 95% CI 0.442–0.788; aHR 0.730, 95% CI 0.603–0.884, respectively). The E-values for the point estimate and upper confidence limit were 2.780 and 1.853 for empagliflozin and 2.082 and 1.517 for dapagliflozin, respectively. However, when comparing individual SGLT-2is, no significant differences were noted between empagliflozin and dapagliflozin in all iCVE outcomes and all-cause mortality (Table 2). The cumulative incidences of iCVE outcomes and all-cause mortality are shown in Figure 2. In the subgroup analysis, consistent results with the main analysis were observed, except for the dapagliflozin group with an aDCSI score of 2 (Supplementary Table S4).

**TABLE 2:** Comparative risk of iCVE outcomes between empagliflozin, dapagliflozin, and dipeptidyl peptidase-4 inhibitors.

iCVE outcomes^a^	No.	Event	IR (per 100 PY)	SGLT-2is vs DPP-4is^b^		Empagliflozin vs dapagliflozin	
				Crude HR (95% CI)	Adjusted HR^c^ (95% CI)	Crude HR (95% CI)	Adjusted HR^c^ (95% CI)
Composite iCVEs^d^							
Empagliflozin	21,304	49	0.14	0.849 (0.639–1.129)	0.925 (0.694–1.233)	0.956 (0.675–1.352)	0.908 (0.641–1.286)
Dapagliflozin	32,563	92	0.15	0.889 (0.719–1.098)	1.019 (0.823–1.262)	Reference	Reference
DPP-4is	419,202	1,480	0.17	Reference	Reference		
Myocardial infarction							
Empagliflozin	21,304	13	0.04	0.587 (0.339–1.017)	0.664 (0.382–1.155)	0.656 (0.348–1.237)	0.619 (0.328–1.169)
Dapagliflozin	32,563	36	0.06	0.894 (0.639–1.253)	1.073 (0.763–1.507)	Reference	Reference
DPP-4is	419,202	586	0.07	Reference	Reference		
Unstable angina							
Empagliflozin	21,304	36	0.11	1.008 (0.722–1.408)	1.073 (0.766–1.502)	1.146 (0.753–1.744)	1.093 (0.717–1.665)
Dapagliflozin	32,563	56	0.09	0.880 (0.670–1.156)	0.981 (0.745–1.292)	Reference	Reference
DPP-4is	419,202	899	0.10	Reference	Reference		
Ischemic stroke							
Empagliflozin	21,304	37	0.11	**0.458 (0.329–0.636)**	**0.568 (0.408–0.791)**	1.007 (0.669–1.514)	0.929 (0.617–1.398)
Dapagliflozin	32,563	64	0.10	**0.455 (0.354–0.583)**	**0.612 (0.476–0.786)**	Reference	Reference
DPP-4is	419,202	2,042	0.23	Reference	Reference		
All-cause mortality							
Empagliflozin	21,304	49	0.14	**0.371 (0.278–0.494)**	**0.590 (0.442–0.788)**	0.846 (0.601–1.190)	0.809 (0.574–1.138)
Dapagliflozin	32,563	112	0.18	**0.438 (0.363–0.530)**	**0.730 (0.603–0.884)**	Reference	Reference
DPP-4is	419,202	3,780	0.42	Reference	Reference		

The incidence rate was the number of events per 100 person-years. Bold data indicate significant estimates.

Abbreviations: iCVE, ischemic cardiovascular event; IR, incidence rate; PY, person-year; SGLT-2is, sodium–glucose cotransporter-2 inhibitors; DPP-4is, dipeptidyl peptidase-4 inhibitors; HR, hazard ratio; CI, confidence interval; CCI, Charlson comorbidity index; aDCSI, adjusted Diabetes Complications Severity Index.

^a^ The incidence of coronary revascularization in both empagliflozin and dapagliflozin groups was zero; thus, it was unable to estimate the hazard ratio between the study groups.

^b^ The DPP-4is group included patients who were newly prescribed alogliptin, anagliptin, evogliptin, teneligliptin, linagliptin, saxagliptin, sitagliptin, vildagliptin, or gemigliptin as an initial or add-on medication.

^c^ Adjusted for age, sex, calendar index year, household income, region of residence, CCI score, comorbidities (hypertension, dyslipidemia, atrial fibrillation, chronic kidney disease, diabetic retinopathy, diabetic neuropathy, diabetic nephropathy, and rheumatoid arthritis), co-medications (antihypertensive agents, antihyperlipidemic agents, antiplatelet agents, anticoagulant agents, and antidiabetic agents), body mass index, smoking status, and aDCSI.

^d^ Composite iCVEs include myocardial infarction, unstable angina, or coronary revascularization.

### 3.3 Effects on safety outcomes

The comparative effects on safety outcomes between individual SGLT-2is and DPP-4is are shown in Table 3. New users of empagliflozin and dapagliflozin showed a significantly lower risk of developing bone fracture than DPP-4is (aHR 0.840, 95% CI 0.779–0.907; aHR 0.944, 95% CI 0.892–1.000, respectively). Moreover, they were observed to have significantly lower risks of UTI (aHR 0.898, 95% CI 0.845–0.956; aHR 0.884, 95% CI 0.841–0.929), severe hypoglycemia (aHR 0.741, 95% CI 0.553–0.992; aHR 0.796, 95% CI 0.640–0.990), and AKI (aHR 0.407, 95% CI 0.244–0.679; aHR 0.261, 95% CI 0.157–0.436) than DPP-4is, respectively. In contrast, for genital infection, both the empagliflozin and dapagliflozin groups showed a significantly higher risk than the DPP-4i group (aHR 2.381, 95% CI 2.262–2.506; aHR 2.349, 95% CI 2.254–2.449). The risk of developing DKA or hypotension was similar between each SGLT-2i drug and DPP-4is. When comparing empagliflozin and dapagliflozin, the empagliflozin group showed a significantly lower risk of bone fracture than the dapagliflozin group (aHR 0.890, 95% CI 0.811–0.976). However, the risks of other safety outcomes were similar between the empagliflozin and dapagliflozin groups (Table 3).

**TABLE 3:** Comparative risk of safety outcomes between empagliflozin, dapagliflozin, and dipeptidyl peptidase-4 inhibitors.

Safety outcomes	No.	Event	IR (per 100 PY)	SGLT-2is vs DPP-4is^a^		Empagliflozin vs dapagliflozin	
				Crude HR (95% CI)	Adjusted HR^b^ (95% CI)	Crude HR (95% CI)	Adjusted HR^b^ (95% CI)
Bone fracture							
Empagliflozin	21,304	703	3.12	**0.767 (0.712–0.827)**	**0.840 (0.779–0.907)**	**0.905 (0.825–0.992)**	**0.890 (0.811–0.976)**
Dapagliflozin	32,563	1,285	3.38	**0.848 (0.801–0.897)**	**0.944 (0.892–1.000)**	Reference	Reference
DPP-4is	419,202	22,349	3.87	Reference	Reference		
Genital infection							
Empagliflozin	21,304	1,714	7.93	**2.665 (2.535–2.803)**	**2.381 (2.262–2.506)**	1.008 (0.949–1.071)	1.013 (0.954–1.077)
Dapagliflozin	32,563	2,762	7.60	**2.644 (2.538–2.754)**	**2.349 (2.254–2.449)**	Reference	Reference
DPP-4is	419,202	15,659	2.68	Reference	Reference		
Severe hypoglycemia							
Empagliflozin	21,304	47	0.20	**0.568 (0.425–0.759)**	**0.741 (0.553–0.992)**	0.868 (0.608–1.238)	0.931 (0.652–1.329)
Dapagliflozin	32,563	87	0.22	**0.655 (0.528–0.813)**	**0.796 (0.640–0.990)**	Reference	Reference
DPP-4is	419,202	1,845	0.31	Reference	Reference		
Urinary tract infection							
Empagliflozin	21,304	1,074	4.83	**0.930 (0.875–0.989)**	**0.898 (0.845–0.956)**	1.024 (0.949–1.106)	1.016 (0.941–1.097)
Dapagliflozin	32,563	1,724	4.59	**0.908 (0.865–0.954)**	**0.884 (0.841–0.929)**	Reference	Reference
DPP-4is	419,202	27,590	4.84	Reference	Reference		
Diabetic ketoacidosis							
Empagliflozin	21,304	22	0.09	0.691 (0.452–1.056)	0.821 (0.535–1.261)	0.747 (0.450–1.242)	0.802 (0.482–1.334)
Dapagliflozin	32,563	47	0.12	0.924 (0.686–1.246)	1.024 (0.756–1.386)	Reference	Reference
DPP-4is	419,202	677	0.11	Reference	Reference		
Acute kidney injury							
Empagliflozin	21,304	15	0.06	**0.363 (0.218–0.605)**	**0.407 (0.244–0.679)**	1.613 (0.789–3.300)	1.557 (0.761–3.186)
Dapagliflozin	32,563	15	0.04	**0.225 (0.135–0.375)**	**0.261 (0.157–0.436)**	Reference	Reference
DPP-4is	419,202	955	0.16	Reference	Reference		
Hypotension							
Empagliflozin	21,304	14	0.06	0.710 (0.417–1.207)	0.877 (0.513–1.499)	0.891 (0.465–1.705)	0.837 (0.437–1.605)
Dapagliflozin	32,563	27	0.07	0.797 (0.537–1.182)	1.047 (0.702–1.562)	Reference	Reference
DPP-4is	419,202	492	0.08	Reference	Reference		

The incidence rate was the number of events per 100 person-years. Bold data indicate significant estimates.

Abbreviations: IR, incidence rate; PY, person-year; SGLT-2is, sodium–glucose cotransporter-2 inhibitors; DPP-4is, dipeptidyl peptidase-4 inhibitors; HR, hazard ratio; CI, confidence interval; CCI, Charlson comorbidity index; aDCSI, adjusted Diabetes Complications Severity Index.

^a^ The DPP-4is group included patients who were newly prescribed alogliptin, anagliptin, evogliptin, teneligliptin, linagliptin, saxagliptin, sitagliptin, vildagliptin, or gemigliptin as an initial or add-on medication.

^b^ Adjusted for age, sex, calendar index year, household income, region of residence, CCI score, comorbidities (hypertension, dyslipidemia, atrial fibrillation, chronic kidney disease, diabetic retinopathy, diabetic neuropathy, diabetic nephropathy, and rheumatoid arthritis), co-medications (antihypertensive agents, antihyperlipidemic agents, antiplatelet agents, anticoagulant agents, and antidiabetic agents), body mass index, smoking status, and aDCSI.

### 3.4 Sensitivity analyses

All results of the sensitivity analyses for iCVE outcomes were aligned with the results of the main analysis (Supplementary Tables S5, S6). With regard to safety outcomes, no difference was observed in the results of analysis using the subdistribution hazard model. However, in a sensitivity analysis counting outcomes occurring ≥1 year after the index date, dapagliflozin did not show a significantly lower risk of severe hypoglycemia compared with DPP-4is. In addition, bone fracture risk was similar between the empagliflozin and dapagliflozin groups.

## 4 Discussion

This nationwide retrospective cohort study investigated the iCVE effectiveness and safety of initiating empagliflozin or dapagliflozin compared with those of DPP-4is in patients with T2DM without a recent CVE history, along with the comparative effects between empagliflozin and dapagliflozin. In this study, the risks of ischemic stroke and all-cause mortality were significantly reduced in both the empagliflozin and dapagliflozin initiating groups compared with those in the DPP-4i initiating group; however, no significant differences were observed between the two individual SGLT-2is. In addition, compared with the initiation of DPP-4is, the initiation of empagliflozin or dapagliflozin was associated with a significantly decreased risk of bone fracture, UTI, severe hypoglycemia, and AKI. In the comparison between individual SGLT-2is, no significant difference was noted except that the risk of bone fracture was lower in the empagliflozin group than in the dapagliflozin group. The results of the subgroup and sensitivity analyses were generally aligned with the main analysis.

To date, the beneficial effects of SGLT-2is for preventing stroke in patients with T2DM are controversial. Two previous cohort studies, which included data from the United States and Europe, showed no difference in the incidence of stroke with the use of SGLT-2is compared with that of DPP-4is (Persson et al., 2018; Patorno et al., 2022). In contrast, other analyses reported a significantly lower risk of stroke with the use of SGLT-2is than with that of DPP-4is (Kosiborod et al., 2018; Dawwas et al., 2019; Han et al., 2021). In this study, both empagliflozin and dapagliflozin significantly reduced the risk of ischemic stroke by almost 40% in patients with T2DM without a recent history of CVE. These different results could be partly explained by differences in the ethnicity of the study populations or the definitions of stroke used in each study. An analysis from the CVD-REAL 2 multinational cohort study, which compared the risk of CVEs in adult patients with T2DM newly initiated on SGLT-2is with that in those newly initiated on DPP-4is using data from clinical practice from 13 countries in the Asia-Pacific, Middle East, European, and North American regions, reported that the risk of stroke was significantly lower in Korea but not in most other enrolled countries (Kohsaka et al., 2020). In addition, in contrast to previous Western studies, a recent observational study on Korean patients with T2DM aged ≥65 years reported that the SGLT-2i class was significantly associated with a reduced risk of stroke compared with DPP-4is (Han et al., 2021). Furthermore, whereas we defined only ischemic stroke as the outcome, almost all previous studies included both hemorrhagic and ischemic strokes (Kosiborod et al., 2018; Persson et al., 2018; Dawwas et al., 2019; Han et al., 2021; Kashiwagi et al., 2022; Patorno et al., 2022). Hemorrhagic and ischemic strokes arise from different pathophysiological pathways, and the characteristics of ischemic stroke are reported to be different between Asians and Caucasians (Bilić et al., 2009; Bang, 2016).

In the current study, unlike favorable effects on ischemic stroke, neither empagliflozin nor dapagliflozin was associated with a significant reduction in the risk of MI, unstable angina, and their composite iCVEs versus DPP-4is. These results on MI and unstable angina were similar to those from previous observational studies (Persson et al., 2018; Han et al., 2021; Kashiwagi et al., 2022; Patorno et al., 2022). Although an analysis from the CVD-REAL 2 study reported a significantly lower risk of MI in the SGLT-2i group than in the DPP-4i group, subanalyses by country showed that no significant differences in MI risk were observed between the SGLT-2i and DPP-4i groups in most countries, including Korea (Kohsaka et al., 2020). In addition, similarly to our study, where the mean age was 55.9 years, a previous observational study found a nonsignificant reduction in hospitalization for MI with SGLT-2is compared with that with DPP-4is when performed in a subanalysis of patients aged <75 years. However, a significant reduction was observed in those aged ≥75 years without a history of cardiovascular disease (Kashiwagi et al., 2022). Therefore, the benefit of SGLT-2is for these iCVEs may also be influenced by patient characteristics, including age and comorbidities.

With respect to safety outcomes, both empagliflozin and dapagliflozin showed significant differences compared with DPP-4is in reducing the risk of bone fracture, severe hypoglycemia, and AKI. Since a potential bone fracture risk was first suggested in the CANVAS trial, there have been concerns that SGLT-2is may cause bone loss by altering calcium and phosphate homeostasis because of secondary hyperparathyroidism due to increased phosphate reabsorption (Mannucci and Monami, 2017). However, several RCTs and meta-analyses, including the EMPA-REG OUTCOME and DECLARE–TIMI 58 trials, reported that SGLT-2is are not related to increased fracture risk (Tang et al., 2016; Mannucci and Monami, 2017). Given that fragility fractures are more common in patients with T2DM (Epstein and LeRoith, 2008), the beneficial effect of SGLT-2is on reducing fracture risk can be investigated further to determine its clinical significance. In addition, similar to previous studies, SGLT-2i initiation was associated with a higher risk of developing genital infection than DPP-4is in this study, resulting from its mechanism of action that inhibits the reabsorption of glucose from the kidney and may cause additional growth of genital commensal microorganisms (Geerlings et al., 2014; Liu et al., 2017). Meanwhile, in this study, empagliflozin and dapagliflozin showed a significantly lower risk of UTI than DPP-4is. This result is aligned with recent data from several real-world studies and meta-analyses that reported a lack of an increased risk of clinically significant UTI with SGLT-2i use (Wiegley and So, 2022). Therefore, our results on the safety of empagliflozin and dapagliflozin provide further reassurance on the safety of SGLT-2i use in patients with T2DM.

This study has some limitations. Because of the nature of the observational study, misclassification bias may exist, and unknown or unmeasured confounding factors, such as baseline HbA1c, diabetes duration, and drinking patterns, may remain unresolved. To compensate for this, we examined the E-value for ischemic stroke and the result indicates that an unmeasured confounder would need to have an association with both treatment and outcome by a HR of >2.918 and >2.651 to negate the observed study results of empagliflozin and dapagliflozin, respectively. Second, this study did not use a propensity score (PS) matching method but rather used covariate adjustment, a conventional method of adjusting for baseline differences between treatment groups by including all possible relevant patient characteristics in the regression model. Although PS matching is an increasingly popular method to adjust for confounding in observational studies, it is not always superior to conventional covariate adjustment. This is supported by the fact that in a study comparing the performance of conventional covariate adjustment with four common PS matching methods using datasets from four large-scale cardiovascular observational studies, both methods performed well (Elze et al., 2017). Third, the mean follow-up period for this study was relatively short. However, in the large cardiovascular outcome trials of SGLT-2is, reductions in the risks of CVEs were consistently noted early in the trial (e.g., 3–6 months), and these benefits continued throughout the study (Zinman et al., 2015; Wiviott et al., 2019). Moreover, a meta-analysis reported that SGLT-2is showed significantly better effects in reducing the risk of CVE than other antidiabetic drugs, regardless of whether the included studies had short (1 year) or long (3–4 years) follow-up periods (Li et al., 2021). Thus, the comparatively short follow-up periods of this study may not have had a serious impact on the results of the current study. Lastly, this study grouped all DPP-4is users without considering the specific drug types used. Further studies considering the impact of individual DPP-4is on CVE may help to complement the findings of this study.

This study showed beneficial outcomes of SGLT-2is, particularly, empagliflozin and dapagliflozin, for ischemic stroke in Asian T2DM patients who had not experienced any CVE within 1 year before the index date. Although reperfusion therapies for acute ischemic stroke have been developed, prevention remains the best strategy for reducing disease burden (Meschia et al., 2014). Primary prevention is especially crucial, as >76% of strokes are first-time events (Meschia et al., 2014). Given the importance of prevention in ischemic stroke, the results of this study may provide meaningful implications in clinical practice. Future long-term multinational large-scale RCTs are needed to better understand and generalize the results of this study.

## Data Availability

The datasets presented in this article are not readily available because of NHIS’s privacy policy. National Health Insurance Sharing Service (NHISS) allows all of this data for qualified researcher who promises to follow the research ethics with some cost. Requests to access the datasets should be directed to the database of the NHISS (available from: https://nhiss.nhis.or.kr/).
