# Supplementary material for: Comparing ischemic cardiovascular effectiveness and safety between individual SGLT-2 inhibitors and DPP-4 inhibitors in patients with type 2 diabetes: a nationwide population-based cohort study
Source: Front Pharmacol. 2024 Oct 30;15:1443175. doi: 10.3389/fphar.2024.1443175 (PMC11561712; doi:10.3389/fphar.2024.1443175)
Supplement: Supplementary file 1 [file Table1.docx]

Supplementary Material

**Supplementary Table 1. A list of outcomes and comorbidities with corresponding codes**

**Supplementary Table 2. A list of drugs used for covariate with corresponding codes**

**Supplementary Table 3. Adapted diabetes** **complications severity index with corresponding diagnostic codes**

**Supplementary Table 4. Subgroup analysis for composite ischemic cardiovascular events associated with the use of empagliflozin, dapagliflozin, and dipeptidyl peptidase-4 inhibitors**

**Supplementary Table 5. Result of the sensitivity analysis using the subdistribution hazard ratio model**

**Supplementary Table 6. Result of the sensitivity analysis counting outcomes occurred ≥1 year after the index date**

Supplementary Table 1. A list of outcomes and comorbidities with corresponding codes

| **Category** | **Corresponding codes** |
| --- | --- |
| **iCVE outcomes** |  |
| Myocardial infarction | ICD-10 codes I21–I23 |
| Unstable angina | ICD-10 codes I20.0 |
| Coronary revascularization | Procedure codes M6551, M6552, M6553, M6554, M6561, M6562, M6563, M6564, M6565, M6566, M6567, M6571, M6572, O1640, O1641, O1642, O1647, O1648, O1649, OA640, OA641, OA642, OA647, OA648, OA649 |
| Ischemic stroke | ICD-10 codes I63 |
| All-cause mortality | All cases of death excluding unnatural deaths such as suicides and accidental deaths |
| **Safety outcomes** | |
| Bone fracture | ICD-10 codes S12, S22, S32, S42, S52, S62, S72, S82, S92 |
| Genital infection | ICD-10 codes B37.3, N77.1, N76, B37.4, N48.1, N47 |
| Severe hypoglycemia | ICD-10 codes E10.0, E11.0, E12.0, E13.0, E14.0, E16.0-2 |
| Urinary tract infection | ICD-10 codes N10–N12, N13.6, N15.1, N15.9, N16, N20.9, N30, N34, N37.0, N39.0 |
| Diabetic ketoacidosis | ICD-10 codes E10.1, E11.1, E12.1, E13.1, E14.1, E87.2 |
| Acute kidney injury | ICD-10 codes N17 |
| Hypotension | ICD-10 codes I95 |
| **Comorbidities** | |
| Hypertension | ICD-10 codes I10–I15 |
| Dyslipidemia | ICD-10 codes E78 |
| Atrial fibrillation | ICD-10 codes I48 |
| Chronic kidney disease | ICD-10 codes N18 |
| Diabetic retinopathy | ICD-10 codes E10.3, E11.3, E12.3, E13.3, E14.3, H36.0 |
| Diabetic neuropathy | ICD-10 codes E10.4, E11.4, E12.4, E13.4, E14.4, G63.2 |
| Diabetic nephropathy | ICD-10 codes E10.2, E11.2, E12.2, E13.2, E14.2, N08.3 |
| Rheumatoid arthritis | ICD-10 codes M05, M06, M08 |

The study outcomes were defined using primary diagnosis code from inpatient claims or emergency department visit.

Abbreviations: iCVE, ischemic cardiovascular event; ICD-10, International Classification of Diseases 10th revision

**Supplementary Table 2. A list of drugs used for covariate with corresponding codes**

| **Drug class** | | | **Drug name** |
| --- | --- | --- | --- |
| Antihypertensive agents | ACEI | | Benazepril, Captopril, Cilazapril, Enalapril, Enalaprilat, Fosinopril, Lisinopril, Moexipril, Perindopril, Quinapril, Ramipril, Trandolapril, |
|  | ARB | | Azilsartan, Candesartan, Eprosartan, Irbesartan, Losartan, Olmesartan, Telmisartan, Valsartan |
|  | CCB | | Amlodipine, Felodipine, Isradipine, Levamlodipine, Nicardipine, Nifedipine, Nimodipine, Nisoldipine, Diltiazem, Verapamil |
|  | BB | | Acebutolol, Atenolol, Betaxolol, Bisoprolol, Carvedilol, Esmolol, Labetalol, Metoprolol, Nadolol, Nebivolol, Pindolol, Propranolol |
|  | Alpha-blocker | | Doxazosin, Prazosin, Terazosin |
|  | DU | | Furosemide, Bumetanide, Torsemide, Triamterene, Spironolactone, Eplerenone, Hydrochlorothiazide, Chlorthalidone, Indapamide, Metolazone, |
|  | Others | | Aliskiren, Clonidine, Hydralazine, Minoxidil |
| Antihyperlipidemic agents | Statin | | Atorvastatin, Fluvastatin, Lovastatin, Pitavastatin, Pravastatin, Rosuvastatin, Simvastatin, Cerivastatin |
|  | Fibrates | | Fenofibrate, Gemfibrozil, Bezafibrate |
|  | Niacin | | Niacin |
|  | Ezetimibe | | Ezetimibe |
|  | PCSK9 inhibitor | | Alirocumab, Evolocumab |
|  | Resin | | Cholestyramine, Colestipol, Colesevelam |
|  | Omega-3 fatty acid | | Omega-3 fatty acid |
| Anticoagulant agents | Vitamin K antagonist | | Warfarin |
|  | UFH | | Heparin |
|  | LMWH | | Dalteparin, Enoxaparin, Nadroparin |
|  | Direct thrombin inhibitor | | Dabigatran, Argatroban |
|  | Factor Xa inhibitor | | Rivaroxaban, Apixaban, Edoxaban, Fondaparinux |
| Antiplatelet agents | COX inhibitor | | Aspirin, Triflusal, Indobufen |
|  | PDE inhibitor | | Cilostarzol, Dipyridamole |
|  | P2Y12 inhibitor  (ADP receptor inhibitor) | | Clopidogrel, Ticlopidine, Prasugrel, Ticagrelor, Cangrelor |
| Antidiabetic agents | Biguanide | | Metformin |
|  | Sulfonylureas | | Chlorpropamide, Tolazamide, Tolbutamide, Gliclazide, Glimepiride, Glipizide, Glyburide (glibenclamide), |
|  | Thiazolidinediones | | Rosiglitazone, Pioglitazone, Lobeglitazone |
|  | Meglitinide | | Repaglinide, Nateglinide, Mitiglinide |
|  | GLP-1RAs | | Albiglutide, Dulaglutide, Exenatide, Liraglutide, Lixisenatide |
|  | Insulin | Rapid-acting insulin | Aspart, Glulisine, Lispro |
|  |  | Short-acting | Regular (Humulin R, Novolin R, Myxredlin) |
|  |  | Intermediate-acting | NPH (Humulin N, Novolin N) |
|  |  | Long-acting | Detemir (Levemir), Glargine (Basaglar, Lantus, Toujeo, Semglee), Degludec (Tresiba) |
|  |  | Pre-mixed (fixed ratios; commercially prepared) | Aspart protamine and aspart mixture (Novolog mix 70/30), Lispro protamine and lispro mixture (Humalog mix 75/25 and 50/50), NPH and regular mixture (Humulin mix 70/30, Novolin mix 70/30) |

Abbreviations: ACEI, angiotensin-converting enzyme inhibitor; ADP, adenosine diphosphate; ARB, angiotensin receptor blocker; BB, beta-blocker; CCB, calcium channel blocker; COX, cyclooxygenase; DU, diuretic; GLP-1RAs, glucagon–like peptide-1 receptor agonists; LMWH, low molecular weight heparin; PCSK9, proprotein convertase subtilisin/kexin type 9; PDE, phosphodiesterase; UFH, unfractionated heparin

**Supplementary Table 3. Adapted diabetes** **complications severity index with corresponding diagnostic codes**

| **Category** | **Complication** | **KCD-7^a^** | **ICD-9** | **DCSI score^b^** |
| --- | --- | --- | --- | --- |
| **Nephropathy** | Diabetic nephropathy | E112, H1020, H1121, N083 | 250.4 | 1 |
|  | Acute glomerulonephritis | N008, N009, N019, | 580 | 1 |
|  | Chronic glomerulonephritis | N038, N031, N035, N039 | 582 | 1 |
|  | Glomerular disorders in other disease classified | N088 | 581.81 | 1 |
|  | Nephrotic syndrome | N040, N042, N045, N048, N049 | 581 | 1 |
|  | Nephritis/nephropathy | N052, N055, N058, N059 | 583 | 1 |
|  | Chronic renal failure | N189 | 585 | 2 |
|  | Unspecified kidney failure | N19 | 586 | 2 |
|  | Renal insufficiency | N289 | 593.9 | 2 |
| **Neuropathy** | Diabetic neuropathy | E114, G609 | 356.9, 250.6 | 1 |
|  | Cranial nerve palsy | S041, S042, S044 | 951.0, 951.1, 951.3 | 1 |
|  | Mono-neuropathy | G56, G57, G58 | 354.0-355.9 | 1 |
|  | Polyneuropathy | G632 | 357.2 | 1 |
|  | Neuropathic arthropathy | M146 | 713.5 | 1 |
|  | Neurogenic bladder | N319 | 596.54 | 1 |
|  | Gastroparesis/diarrhea | K591, K3188 | 564.5, 536.3 | 1 |
|  | Orthostatic hypotension | I951 | 458.0 | 1 |
| **Retinopathy** | Diabetic ophthalmologic disease | E113 | 250.5x | 1 |
|  | Background retinopathy | H350 | 362.01 | 1 |
|  | Other retinopathy | H360 | 362.1 | 1 |
|  | Retinal edema | H358 | 362.83 | 1 |
|  | CSME (clinically significant macular edema) | H353 | 362.53 | 1 |
|  | Other retinal disorders | H356 | 362.81, 362.82 | 1 |
|  | Vitreous hemorrhage | H431 | 379.23 | 1 |
|  | Retinal detachment | H33 | 361.xx | 2 |
|  | Blindness | H540 | 369.xx .00-.99 | 2 |
| **Cerebrovascular disease^c^** | Transient cerebral ischemic attacks | G45 | 435 | 1 |
|  | Stroke | I61, I63, I64 | 431, 433, 434, 436 | 2 |
| **Cardiovascular disease^c^** | Atherosclerosis | I70 | 440.xx | 1 |
|  | Angina pectoris | I20 | 413 | 1 |
|  | Other acute ischemic heart diseases | I24 | 411 | 1 |
|  | Chronic ischemic heart diseases | I25 | 414 | 1 |
|  | Myocardial infarction | I21 | 410 | 2 |
|  | Ventricular fibrillation, arrest | I472 | 427.1, 427.3 | 2 |
|  | Atrial fibrillation, arrest | I48, I49, I46 | 427.4, 427.5 | 2 |
|  | Congestive heart failure | I500 | 428 | 2 |
|  | Aortic aneurysm, dissection | I71 | 441 | 2 |
| **Peripheral vascular disease (PVD)** | Diabetic PVD | E1151, I792 | 250.7 | 1 |
|  | PVD | I739 | 443.81, 443.9 | 1 |
|  | Embolism/thrombosis (LE) | I743 | 444.22 | 2 |
|  | Other aneurysm, lower extreme | I72 | 442.3 | 1 |
|  | Gangrene | R02 | 785.4 | 2 |
|  | Ulcer of lower limbs | L97 | 707.1 | 2 |
| **Acute metabolic complications** | Diabetes with ketoacidosis | E1110 | 250.1 | 2 |
|  | Diabetes with hyperosmolar | E1100 | 250.2 | 2 |
|  | Diabetes with other coma | E1101, E1102, E1103, E1104 | 250.3 | 2 |

The sum of the scores for each complication in seven categories is the overall total aDCSI score. Even if multiple complications were identified in the same category, only one disease type with the highest score is factored into the calculation.

Abbreviations: ICD, International Classification of Diseases; KCD, Korean Standard Classification of Disease; DCSI, Diabetes Complications Severity Index

^a^Validation of aDCSI with Korean Standard Classification of Disease 7 in Korean population were conducted in previous study [1].

^b^The aDCSI score ranges from 0 to 2 and indicates the severity of complications of diabetes as follows: not present or normal = 0; some abnormality = 1; 2 = severe abnormality [2].

^c^Cerebrovascular and cardiovascular diseases were excluded when scoring the aDCSI score in this study because they overlapped with the current study outcomes; thus, the total modified score ranged from 0 to 9.

**Supplementary Table 4. Subgroup analysis for composite ischemic cardiovascular events associated with use of empagliflozin, dapagliflozin, and dipeptidyl peptidase-4 inhibitors**

| **Variable** | **Study group** | **No.** | **Event** | **IR (per 100 PY)** | **Crude HR (95% CI)** | **Adjusted HR^a^ (95% CI)** |
| --- | --- | --- | --- | --- | --- | --- |
| **Age (year)** | | | | | | |
| <65 | Empagliflozin | 18,608 | 35 | 0.12 | 0.837 (0.597–1.174) | 0.855 (0.609–1.201) |
|  | Dapagliflozin | 29,169 | 80 | 0.14 | 1.052 (0.837–1.321) | 1.112 (0.883–1.400) |
|  | DPP-4is | 319,577 | 947 | 0.14 | Reference | Reference |
| 65–74 | Empagliflozin | 2,212 | 13 | 0.38 | 1.557 (0.895–2.710) | 1.507 (0.862–2.632) |
|  | Dapagliflozin | 2,838 | 9 | 0.17 | 0.637 (0.316–1.285) | 0.635 (0.315–1.282) |
|  | DPP-4is | 71,599 | 353 | 0.24 | Reference | Reference |
| ≥75 | Empagliflozin | 484 | 1 | 0.14 | 0.422 (0.059–3.008) | 0.374 (0.052–2.677) |
|  | Dapagliflozin | 556 | 3 | 0.30 | 0.939 (0.300–2.939) | 0.847 (0.270–2.659) |
|  | DPP-4is | 28,026 | 180 | 0.32 | Reference | Reference |
| **Sex** | | | | | | |
| Male | Empagliflozin | 12,566 | 36 | 0.18 | 0.908 (0.651–1.267) | 0.962 (0.688–1.345) |
|  | Dapagliflozin | 19,380 | 74 | 0.21 | 1.038 (0.819–1.317) | 1.150 (0.905–1.462) |
|  | DPP-4is | 256,990 | 1,071 | 0.20 | Reference | Reference |
| Female | Empagliflozin | 8,738 | 13 | 0.09 | 0.749 (0.431–1.302) | 0.843 (0.483–1.472) |
|  | Dapagliflozin | 13,183 | 18 | 0.07 | 0.589 (0.367–0.944) | 0.707 (0.439–1.139) |
|  | DPP-4is | 162,212 | 409 | 0.12 | Reference | Reference |
| **BMI (kg/m^2^)** | | | | | | |
| 18–24.9 | Empagliflozin | 5,046 | 16 | 0.20 | 1.120 (0.681–1.840) | 1.154 (0.701–1.901) |
|  | Dapagliflozin | 7,311 | 15 | 0.11 | 0.618 (0.370–1.031) | 0.679 (0.407–1.135) |
|  | DPP-4is | 167,190 | 616 | 0.17 | Reference | Reference |
| 25–29.9 | Empagliflozin | 10,402 | 24 | 0.15 | 0.823 (0.547–1.236) | 0.874 (0.580–1.317) |
|  | Dapagliflozin | 15,872 | 55 | 0.18 | 1.049 (0.795–1.383) | 1.169 (0.885–1.545) |
|  | DPP-4is | 193,094 | 707 | 0.17 | Reference | Reference |
| ≥30 | Empagliflozin | 5,856 | 9 | 0.10 | 0.730 (0.372–1.429) | 0.781 (0.397–1.539) |
|  | Dapagliflozin | 9,380 | 22 | 0.12 | 0.980 (0.627–1.532) | 1.054 (0.672–1.653) |
|  | DPP-4is | 58,918 | 157 | 0.13 | Reference | Reference |
| **eGFR (mL/min/1.73 m^2^)**^b^ | | | | | | |
| <60 | Empagliflozin | 745 | 3 | 0.26 | 0.937 (0.298–2.947) | 0.927 (0.292–2.940) |
|  | Dapagliflozin | 929 | 2 | 0.12 | 0.418 (0.103–1.689) | 0.476 (0.117–1.934) |
|  | DPP-4is | 21,309 | 126 | 0.28 | Reference | Reference |
| 60–89.9 | Empagliflozin | 9,252 | 28 | 0.19 | 1.000 (0.685–1.459) | 1.095 (0.748–1.602) |
|  | Dapagliflozin | 13,869 | 45 | 0.17 | 0.926 (0.685–1.250) | 1.028 (0.759–1.393) |
|  | DPP-4is | 191,723 | 761 | 0.19 | Reference | Reference |
| ≥90 | Empagliflozin | 11,193 | 17 | 0.09 | 0.698 (0.431–1.131) | 0.747 (0.460–1.215) |
|  | Dapagliflozin | 17,605 | 45 | 0.13 | 0.991 (0.729–1.347) | 1.127 (0.827–1.537) |
|  | DPP-4is | 202,247 | 570 | 0.13 | Reference | Reference |
| **LDL-C (mg/dL)**^b^ | | | | | | |
| <70 | Empagliflozin | 2,373 | 8 | 0.18 | 0.821 (0.406–1.662) | 0.831 (0.408–1.692) |
|  | Dapagliflozin | 3,485 | 18 | 0.23 | 1.097 (0.680–1.772) | 1.160 (0.715–1.883) |
|  | DPP-4is | 47,545 | 241 | 0.21 | Reference | Reference |
| 70–99.9 | Empagliflozin | 3,363 | 12 | 0.19 | 1.101 (0.618–1.962) | 1.145 (0.639–2.052) |
|  | Dapagliflozin | 5,389 | 15 | 0.12 | 0.740 (0.440–1.242) | 0.817 (0.484–1.378) |
|  | DPP-4is | 74,172 | 303 | 0.17 | Reference | Reference |
| 100–129.9 | Empagliflozin | 3,386 | 12 | 0.19 | 1.191 (0.669–2.123) | 1.337 (0.746–2.398) |
|  | Dapagliflozin | 5,570 | 19 | 0.15 | 0.938 (0.583–1.510) | 1.125 (0.695–1.820) |
|  | DPP-4is | 79,573 | 296 | 0.15 | Reference | Reference |
| 130–159.9 | Empagliflozin | 2,322 | 3 | 0.07 | 0.437 (0.140–1.365) | 0.510 (0.162–1.601) |
|  | Dapagliflozin | 4,054 | 14 | 0.15 | 1.014 (0.590–1.742) | 1.194 (0.690–2.067) |
|  | DPP-4is | 56,772 | 210 | 0.15 | Reference | Reference |
| ≥160 | Empagliflozin | 1,561 | 2 | 0.07 | 0.421 (0.104–1.702) | 0.455 (0.112–1.847) |
|  | Dapagliflozin | 2,654 | 8 | 0.14 | 0.890 (0.436–1.815) | 1.035 (0.504–2.125) |
|  | DPP-4is | 37,020 | 138 | 0.15 | Reference | Reference |
| **Antiplatelet agents use** | | | | | | |
| Yes | Empagliflozin | 2,626 | 20 | 0.48 | 1.213 (0.773–1.902) | 1.258 (0.798–1.984) |
|  | Dapagliflozin | 3,424 | 33 | 0.51 | 1.280 (0.892–1.837) | 1.360 (0.943–1.960) |
|  | DPP-4is | 46,728 | 380 | 0.39 | Reference | Reference |
| No | Empagliflozin | 18,678 | 29 | 0.10 | 0.688 (0.476–0.995) | 0.780 (0.538–1.130) |
|  | Dapagliflozin | 29,139 | 59 | 0.11 | 0.771 (0.593–1.002) | 0.892 (0.685–1.162) |
|  | DPP-4is | 372,474 | 1,100 | 0.14 | Reference | Reference |
| **Insulin use** | | | | | | |
| Yes | Empagliflozin | 282 | 0 | 0.00 | 0.000 | 0.000 |
|  | Dapagliflozin | 406 | 1 | 0.12 | 0.664 (0.086–5.107) | 1.300 (0.154–10.980) |
|  | DPP-4is | 2,882 | 12 | 0.18 | Reference | Reference |
| No | Empagliflozin | 21,022 | 49 | 0.15 | 0.862 (0.648–1.146) | 0.937 (0.703–1.249) |
|  | Dapagliflozin | 32,157 | 91 | 0.15 | 0.891 (0.720–1.103) | 1.019 (0.822–1.264) |
|  | DPP-4is | 416,320 | 1,468 | 0.17 | Reference | Reference |
| **PDC ≥80%** | | | | | | |
| Yes | Empagliflozin | 18,831 | 48 | 0.16 | 0.816 (0.612–1.088) | 0.900 (0.674–1.203) |
|  | Dapagliflozin | 28,876 | 89 | 0.16 | 0.843 (0.680–1.045) | 0.971 (0.781–1.207) |
|  | DPP-4is | 357,897 | 1,448 | 0.19 | Reference | Reference |
| No | Empagliflozin | 2,473 | 1 | 0.03 | 1.131 (0.154–8.312) | 0.986 (0.132–7.356) |
|  | Dapagliflozin | 3,687 | 3 | 0.04 | 1.858 (0.568–6.081) | 1.865 (0.552–6.293) |
|  | DPP-4is | 61,305 | 32 | 0.02 | Reference | Reference |
| **aDCSI score** | | | | | | |
| 0 | Empagliflozin | 12,440 | 31 | 0.16 | 1.095 (0.764–1.569) | 1.165 (0.810–1.676) |
|  | Dapagliflozin | 19,061 | 53 | 0.15 | 1.021 (0.771–1.353) | 1.149 (0.864–1.527) |
|  | DPP-4is | 240,149 | 726 | 0.14 | Reference | Reference |
| 1 | Empagliflozin | 6,240 | 12 | 0.12 | 0.641 (0.361–1.137) | 0.712 (0.400–1.268) |
|  | Dapagliflozin | 9,626 | 32 | 0.17 | 0.948 (0.663–1.356) | 1.098 (0.765–1.576) |
|  | DPP-4is | 122,885 | 483 | 0.19 | Reference | Reference |
| 2 | Empagliflozin | 2,093 | 6 | 0.18 | 0.706 (0.314–1.590) | 0.763 (0.337–1.728) |
|  | Dapagliflozin | 3,139 | 5 | 0.09 | 0.360 (0.148–0.873) | **0.406 (0.166–0.989)** |
|  | DPP-4is | 43,836 | 215 | 0.23 | Reference | Reference |
| 3 | Empagliflozin | 455 | 0 | 0.00 | 0.000 | 0.000 |
|  | Dapagliflozin | 628 | 2 | 0.16 | 0.709 (0.172–2.916) | 0.823 (0.196–3.453) |
|  | DPP-4is | 10,297 | 49 | 0.23 | Reference | Reference |
| 4 | Empagliflozin | 67 | 0 | 0.00 | NA | NA |
|  | Dapagliflozin | 98 | 0 | 0.00 | NA | NA |
|  | DPP-4is | 1,787 | 7 | 0.20 | Reference | Reference |
| 5 | Empagliflozin | 9 | 0 | 0.00 | NA | NA |
|  | Dapagliflozin | 11 | 0 | 0.00 | NA | NA |
|  | DPP-4is | 229 | 0 | 0.00 | Reference | Reference |
| 6 | Empagliflozin | . | . | NA | NA | NA |
|  | Dapagliflozin | . | . | NA | NA | NA |
|  | DPP-4is | 19 | 0 | 0.00 | Reference | Reference |

Composite iCVEs include myocardial infection, unstable angina, or coronary revascularization.

The incidence rate was the number of events per 100 person-years. Bold data indicate significant estimates.

Abbreviations: iCVE, ischemic cardiovascular event; DPP-4is, dipeptidyl peptidase-4 inhibitors; IR, incidence rate; PY, person-year; HR, hazard ratio; CI, confidence interval; eGFR, estimated glomerular filtration rate; LDL-C, low-density lipoprotein-cholesterol; PDC, proportion of days covered; aDCSI, adjusted Diabetes Complications Severity Index; NA, not addressed

^a^Adjusted for age, sex, calendar index year, household income, region of residence, CCI score, comorbidities (hypertension, dyslipidemia, atrial fibrillation, chronic kidney disease, diabetic retinopathy, diabetic neuropathy, diabetic nephropathy, and rheumatoid arthritis), co-medications (antihypertensive agents, antihyperlipidemic agents, antiplatelet agents, anticoagulant agents, and antidiabetic agents), body mass index, smoking status, and aDCSI.

^b^The percentages within each column do not add up to 100% because some data are unavailable.

**Supplementary Table 5. Result of the sensitivity analysis using the subdistribution hazard ratio model**

**(A) iCVE outcomes**

| **iCVE outcomes^a^** | **No.** | **Event** | **IR (per 100 PY)** | **SGLT-2is vs. DPP-4is** | | **Empagliflozin vs. dapagliflozin** | |
| --- | --- | --- | --- | --- | --- | --- | --- |
|  |  |  |  | **Crude HR (95% CI)** | **Adjusted HR^b^ (95% CI)** | **Crude HR (95% CI)** | **Adjusted HR^b^ (95% CI)** |
| **Composite iCVEs^c^** | | | | | | | |
| Empagliflozin | 21,304 | 49 | 0.14 | 0.849 (0.639–1.129) | 0.925 (0.694–1.233) | 0.956 (0.675–1.353) | 0.908 (0.641–1.285) |
| Dapagliflozin | 32,563 | 92 | 0.15 | 0.889 (0.719–1.098) | 1.019 (0.823–1.261) | Reference | Reference |
| DPP-4is | 419,202 | 1,480 | 0.17 | Reference | Reference |  |  |
| **Myocardial infarction** | | | | | | | |
| Empagliflozin | 21,304 | 13 | 0.04 | 0.587 (0.338–1.017) | 0.664 (0.383–1.153) | 0.656 (0.348–1.238) | 0.619 (0.328–1.168) |
| Dapagliflozin | 32,563 | 36 | 0.06 | 0.894 (0.639–1.252) | 1.073 (0.765–1.503) | Reference | Reference |
| DPP-4is | 419,202 | 586 | 0.07 | Reference | Reference |  |  |
| **Unstable angina** | | | | | | | |
| Empagliflozin | 21,304 | 36 | 0.11 | 1.008 (0.723–1.407) | 1.073 (0.765–1.504) | 1.146 (0.753–1.744) | 1.093 (0.718–1.664) |
| Dapagliflozin | 32,563 | 56 | 0.09 | 0.880 (0.670–1.156) | 0.981 (0.746–1.291) | Reference | Reference |
| DPP-4is | 419,202 | 899 | 0.10 | Reference | Reference |  |  |
| **Ischemic stroke** | | | | | | | |
| Empagliflozin | 21,304 | 37 | 0.11 | **0.458 (0.329–0.636)** | **0.568 (0.408–0.791)** | 1.007 (0.669–1.514) | 0.929 (0.617–1.397) |
| Dapagliflozin | 32,563 | 64 | 0.10 | **0.455 (0.355–0.583)** | **0.612 (0.476–0.786)** | Reference | Reference |
| DPP-4is | 419,202 | 2,042 | 0.23 | Reference | Reference |  |  |
| **All-cause mortality** | | | | | | | |
| Empagliflozin | 21,304 | 49 | 0.14 | **0.371 (0.278–0.494)** | **0.590 (0.442–0.789)** | 0.846 (0.601–1.190) | 0.809 (0.574–1.138) |
| Dapagliflozin | 32,563 | 112 | 0.18 | **0.438 (0.363–0.530)** | **0.730 (0.603–0.884)** | Reference | Reference |
| DPP-4is | 419,202 | 3,780 | 0.42 | Reference | Reference |  |  |

The incidence rate was the number of events per 100 person-years. Bold data indicate significant estimates.

Abbreviations: iCVE, ischemic cardiovascular event; IR, incidence rate; PY, person-year; SGLT-2is, sodium–glucose cotransporter-2 inhibitors; DPP-4is, dipeptidyl peptidase-4 inhibitors; HR, hazard ratio; CI, confidence interval; CCI, Charlson comorbidity index; aDCSI, adjusted Diabetes Complications Severity Index

^a^The incidence of coronary revascularization in both empagliflozin and dapagliflozin groups was zero; thus, it was unable to estimate the hazard ratio between the study groups.

^b^Adjusted for age, sex, calendar index year, household income, region of residence, CCI score, comorbidities (hypertension, dyslipidemia, atrial fibrillation, chronic kidney disease, diabetic retinopathy, diabetic neuropathy, diabetic nephropathy, and rheumatoid arthritis), co-medications (antihypertensive agents, antihyperlipidemic agents, antiplatelet agents, anticoagulant agents, and antidiabetic agents), body mass index, smoking status, and aDCSI.

^c^Composite iCVEs include myocardial infarction, unstable angina, or coronary revascularization.

**(B) Safety outcomes**

| **Safety outcomes** | **No.** | **Event** | **IR (per 100 PY)** | **SGLT-2is vs. DPP-4is** | | **Empagliflozin vs. dapagliflozin** | |
| --- | --- | --- | --- | --- | --- | --- | --- |
|  |  |  |  | **Crude HR (95% CI)** | **Adjusted HR^a^ (95% CI)** | **Crude HR (95% CI)** | **Adjusted HR^a^ (95% CI)** |
| **Bone fracture** | | | | | | | |
| Empagliflozin | 21,304 | 703 | 3.12 | **0.770 (0.714**–**0.830)** | **0.841 (0.780**–**0.907)** | **0.904 (0.825**–**0.992)** | **0.891 (0.812**–**0.977)** |
| Dapagliflozin | 32,563 | 1,285 | 3.38 | **0.851 (0.804**–**0.900)** | **0.944 (0.892**–**0.999)** | Reference | Reference |
| DPP-4is | 419,202 | 22,349 | 3.87 | Reference | Reference |  |  |
| **Genital infection** | | | | | | | |
| Empagliflozin | 21,304 | 1,714 | 7.93 | **2.673 (2.542***–***2.811)** | **2.383 (2.263–2.508)** | 1.008 (0.949–1.071) | 1.014 (0.953–1.078) |
| Dapagliflozin | 32,563 | 2,762 | 7.60 | **2.652 (2.546–2.762)** | **2.350 (2.254–2.451)** | Reference | Reference |
| DPP-4is | 419,202 | 15,659 | 2.68 | Reference | Reference |  |  |
| **Severe hypoglycemia** | | | | | | | |
| Empagliflozin | 21,304 | 47 | 0.20 | **0.569 (0.426–0.761)** | **0.741 (0.554–0.992)** | 0.867 (0.608–1.238) | 0.932 (0.653–1.330) |
| Dapagliflozin | 32,563 | 87 | 0.22 | **0.656 (0.529–0.815)** | **0.796 (0.639–0.991)** | Reference | Reference |
| DPP-4is | 419,202 | 1,845 | 0.31 | Reference | Reference |  |  |
| **Urinary tract infection** | | | | | | | |
| Empagliflozin | 21,304 | 1,074 | 4.83 | **0.933 (0.878**–**0.992)** | **0.899 (0.845–0.957)** | 1.024 (0.949–1.106) | 1.017 (0.942–1.098) |
| Dapagliflozin | 32,563 | 1,724 | 4.59 | **0.911 (0.868–0.957)** | **0.884 (0.841–0.929)** | Reference | Reference |
| DPP-4is | 419,202 | 27,590 | 4.84 | Reference | Reference |  |  |
| **Diabetic ketoacidosis** | | | | | | | |
| Empagliflozin | 21,304 | 22 | 0.09 | 0.822 (0.535–1.261) | 0.692 (0.452–1.057) | 0.747 (0.450–1.242) | 0.802 (0.483–1.332) |
| Dapagliflozin | 32,563 | 47 | 0.12 | 1.024 (0.756–1.386) | 0.925 (0.686–1.248) | Reference | Reference |
| DPP-4is | 419,202 | 677 | 0.11 | Reference | Reference |  |  |
| **Acute kidney injury** | | | | | | | |
| Empagliflozin | 21,304 | 15 | 0.06 | **0.364 (0.219–0.606)** | **0.407 (0.244–0.678)** | 1.613 (0.788–3.299) | 1.558 (0.761–3.188) |
| Dapagliflozin | 32,563 | 15 | 0.04 | **0.226 (0.136–0.376)** | **0.261 (0.156–0.436)** | Reference | Reference |
| DPP-4is | 419,202 | 955 | 0.16 | Reference | Reference |  |  |
| **Hypotension** | | | | | | | |
| Empagliflozin | 21,304 | 14 | 0.06 | 0.712 (0.418–1.211) | 0.878 (0.511–1.509) | 0.890 (0.465–1.704) | 0.839 (0.437–1.610) |
| Dapagliflozin | 32,563 | 27 | 0.07 | 0.800 (0.539–1.186) | 1.047 (0.700–1.564) | Reference | Reference |
| DPP-4is | 419,202 | 492 | 0.08 | Reference | Reference |  |  |

The incidence rate was the number of events per 100 person-years. Bold data indicate significant estimates.

Abbreviations: IR, incidence rate; PY, person-year; SGLT-2is, sodium–glucose cotransporter-2 inhibitors; DPP-4is, dipeptidyl peptidase-4 inhibitors; HR, hazard ratio; CI, confidence interval; CCI, Charlson comorbidity index; aDCSI, adjusted Diabetes Complications Severity Index

^a^Adjusted for age, sex, calendar index year, household income, region of residence, CCI score, comorbidities (hypertension, dyslipidemia, atrial fibrillation, chronic kidney disease, diabetic retinopathy, diabetic neuropathy, diabetic nephropathy, and rheumatoid arthritis), co-medications (antihypertensive agents, antihyperlipidemic agents, antiplatelet agents, anticoagulant agents, and antidiabetic agents), body mass index, smoking status, and aDCSI.

**Supplementary Table 6. Result of the sensitivity analysis counting outcomes occurred ≥1 year after the index date**

**(A) iCVE outcomes**

| **iCVE outcomes^a^** | **No.** | **Event** | **IR (per 100 PY)** | **SGLT-2is vs. DPP-4is** | | **Empagliflozin vs. dapagliflozin** | |
| --- | --- | --- | --- | --- | --- | --- | --- |
|  |  |  |  | **Crude HR (95% CI)** | **Adjusted HR^b^ (95% CI)** | **Crude HR (95% CI)** | **Adjusted HR^b^ (95% CI)** |
| **Composite iCVEs^c^** | | | | | | | |
| Empagliflozin | 21,304 | 24 | 0.07 | 0.916 (0.610–1.375) | 1.013 (0.672–1.526) | 1.052 (0.641–1.727) | 1.001 (0.609–1.645) |
| Dapagliflozin | 32,563 | 45 | 0.07 | 0.871 (0.645–1.176) | 1.012 (0.747–1.370) | Reference | Reference |
| DPP-4is | 419,202 | 790 | 0.09 | Reference | Reference |  |  |
| **Myocardial infarction** | | | | | | | |
| Empagliflozin | 21,304 | 7 | 0.02 | 0.645 (0.305–1.365) | 0.751 (0.353–1.594) | 0.951 (0.388–2.333) | 0.902 (0.367–2.215) |
| Dapagliflozin | 32,563 | 15 | 0.02 | 0.678 (0.404–1.138) | 0.832 (0.494–1.402) | Reference | Reference |
| DPP-4is | 419,202 | 337 | 0.04 | Reference | Reference |  |  |
| **Unstable angina** | | | | | | | |
| Empagliflozin | 21,304 | 17 | 0.05 | 1.098 (0.676–1.782) | 1.172 (0.719–1.912) | 1.094 (0.603–1.983) | 1.043 (0.574–1.893) |
| Dapagliflozin | 32,563 | 30 | 0.05 | 1.003 (0.693–1.452) | 1.124 (0.774–1.633) | Reference | Reference |
| DPP-4is | 419,202 | 458 | 0.05 | Reference | Reference |  |  |
| **Ischemic stroke** | | | | | | | |
| Empagliflozin | 21,304 | 16 | 0.05 | **0.405 (0.243–0.675)** | **0.523 (0.313–0.872)** | 1.149 (0.609–2.169) | 1.070 (0.566–2.021) |
| Dapagliflozin | 32,563 | 26 | 0.04 | **0.353 (0.239–0.520)** | **0.489 (0.331–0.722)** | Reference | Reference |
| DPP-4is | 419,202 | 1,131 | 0.13 | Reference | Reference |  |  |
| **All-cause mortality** | | | | | | | |
| Empagliflozin | 21,304 | 49 | 0.14 | **0.371 (0.278–0.494)** | **0.590 (0.442–0.788)** | 0.846 (0.601–1.190) | 0.809 (0.574–1.138) |
| Dapagliflozin | 32,563 | 112 | 0.18 | **0.438 (0.363–0.530)** | **0.730 (0.603–0.884)** | Reference | Reference |
| DPP-4is | 419,202 | 3,780 | 0.42 | Reference | Reference |  |  |

The incidence rate was the number of events per 100 person-years. Bold data indicate significant estimates.

Abbreviations: iCVE, ischemic cardiovascular disease; IR, incidence rate; PY, person-year; SGLT-2is, sodium–glucose cotransporter-2 inhibitors; DPP-4is, dipeptidyl peptidase-4 inhibitors; HR, hazard ratio; CI, confidence interval; CCI, Charlson comorbidity index; aDCSI, adjusted Diabetes Complications Severity Index;

^a^The incidence of coronary revascularization in both empagliflozin and dapagliflozin groups was zero; thus, it was unable to estimate the hazard ratio between the study groups.

^b^Adjusted for age, sex, calendar index year, household income, region of residence, CCI score, comorbidities (hypertension, dyslipidemia, atrial fibrillation, chronic kidney disease, diabetic retinopathy, diabetic neuropathy, diabetic nephropathy, and rheumatoid arthritis), co-medications (antihypertensive agents, antihyperlipidemic agents, antiplatelet agents, anticoagulant agents, and antidiabetic agents), body mass index, smoking status, and aDCSI.

^c^Composite iCVEs include myocardial infarction, unstable angina, or coronary revascularization.

**(B) Safety outcomes**

| **Safety outcomes** | **No.** | **Event** | **IR (per 100 PY)** | **SGLT-2is vs. DPP-4is** | | **Empagliflozin vs. dapagliflozin** | |
| --- | --- | --- | --- | --- | --- | --- | --- |
|  |  |  |  | **Crude HR (95% CI)** | **Adjusted HR^a^ (95% CI)** | **Crude HR (95% CI)** | **Adjusted HR^a^ (95% CI)** |
| **Bone fracture** | | | | | | | |
| Empagliflozin | 21,304 | 511 | 2.26 | **0.750 (0.686**–**0.819)** | **0.876 (0.801**–**0.957)** | 0.938 (0.842–1.046) | 0.964 (0.865–1.074) |
| Dapagliflozin | 32,563 | 921 | 2.42 | **0.799 (0.748**–**0.854)** | **0.909 (0.850**–**0.972)** | Reference | Reference |
| DPP-4is | 419,202 | 17,649 | 3.06 | Reference | Reference |  |  |
| **Genital infection** | | | | | | | |
| Empagliflozin | 21,304 | 1,088 | 5.03 | **2.249 (2.113**–**2.393)** | **2.192 (2.057**–**2.335)** | 0.969 (0.899–1.045) | 1.022 (0.948–1.102) |
| Dapagliflozin | 32,563 | 1,852 | 5.10 | **2.320 (2.209**–**2.437)** | **2.144 (2.040**–**2.254)** | Reference | Reference |
| DPP-4is | 419,202 | 12,470 | 2.13 | Reference | Reference |  |  |
| **Severe hypoglycemia** | | | | | | | |
| Empagliflozin | 21,304 | 24 | 0.10 | **0.445 (0.297**–**0.666)** | **0.642 (0.428**–**0.964)** | 0.733 (0.453–1.188) | 0.838 (0.517–1.358) |
| Dapagliflozin | 32,563 | 53 | 0.14 | **0.607 (0.461**–**0.799)** | 0.766 (0.581–1.011) | Reference | Reference |
| DPP-4is | 419,202 | 1,265 | 0.21 | Reference | Reference |  |  |
| **Urinary tract infection** | | | | | | | |
| Empagliflozin | 21,304 | 734 | 3.30 | **0.828 (0.769**–**0.892)** | **0.855 (0.793**–**0.921)** | 0.972 (0.887–1.065) | 1.009 (0.921–1.106) |
| Dapagliflozin | 32,563 | 1,262 | 3.36 | **0.852 (0.805**–**0.902)** | **0.847 (0.800**–**0.897)** | Reference | Reference |
| DPP-4is | 419,202 | 22,200 | 3.90 | Reference | Reference |  |  |
| **Diabetic ketoacidosis** | | | | | | | |
| Empagliflozin | 21,304 | 13 | 0.06 | 0.776 (0.446–1.350) | 1.121 (0.641–1.960) | 0.984 (0.493–1.966) | 1.132 (0.566–2.263) |
| Dapagliflozin | 32,563 | 21 | 0.05 | 0.789 (0.508–1.224) | 0.990 (0.635–1.545) | Reference | Reference |
| DPP-4is | 419,202 | 375 | 0.06 | Reference | Reference |  |  |
| **Acute kidney injury** | | | | | | | |
| Empagliflozin | 21,304 | 8 | 0.03 | **0.289 (0.144**–**0.580)** | **0.345 (0.171**–**0.694)** | 1.323 (0.522–3.352) | 1.351 (0.533–3.424) |
| Dapagliflozin | 32,563 | 10 | 0.03 | **0.218 (0.117**–**0.408)** | **0.255 (0.136**–**0.477)** | Reference | Reference |
| DPP-4is | 419,202 | 686 | 0.11 | Reference | Reference |  |  |
| **Hypotension** | | | | | | | |
| Empagliflozin | 21,304 | 8 | 0.03 | 0.557 (0.276–1.123) | 0.737 (0.364–1.493) | 0.715 (0.313–1.633) | 0.699 (0.305–1.598) |
| Dapagliflozin | 32,563 | 19 | 0.05 | 0.779 (0.491–1.236) | 1.055 (0.662–1.683) | Reference | Reference |
| DPP-4is | 419,202 | 383 | 0.06 | Reference | Reference |  |  |

The incidence rate was the number of events per 100 person-years. Bold data indicate significant estimates.

Abbreviations: IR, incidence rate; PY, person-year; SGLT-2is, sodium–glucose cotransporter-2 inhibitors; DPP-4is, dipeptidyl peptidase-4 inhibitors; HR, hazard ratio; CI, confidence interval; CCI, Charlson comorbidity index; aDCSI, adjusted Diabetes Complications Severity Index

^a^Adjusted for age, sex, calendar index year, household income, region of residence, CCI score, comorbidities (hypertension, dyslipidemia, atrial fibrillation, chronic kidney disease, diabetic retinopathy, diabetic neuropathy, diabetic nephropathy, and rheumatoid arthritis), co-medications (antihypertensive agents, antihyperlipidemic agents, antiplatelet agents, anticoagulant agents, and antidiabetic agents), body mass index, smoking status, and aDCSI.

**Reference**

1. Yoo H, Choo E, Lee S. Study of hospitalization and mortality in Korean diabetic patients using the diabetes complications severity index. BMC Endocr Disord 2020;20:122.
2. Young BA, Lin E, Von Korff M, Simon G, Ciechanowski P, Ludman EJ, et al. Diabetes complications severity index and risk of mortality, hospitalization, and healthcare utilization. Am J Manag Care 2008;14:15-23.
